# Proteomic analyses of sheep (*ovis aries*) embryonic skeletal muscle

**DOI:** 10.1038/s41598-020-58349-0

**Published:** 2020-02-04

**Authors:** Xinyue Wang, Tianpei Shi, Zhida Zhao, Haobin Hou, Li Zhang

**Affiliations:** Institute of Animal Science, Chinese Academy of Agricultural Sciences, (CAAS), Beijing, China

**Keywords:** Proteomic analysis, Animal breeding

## Abstract

The growth and development of embryonic skeletal muscle plays a crucial role in sheep muscle mass. But proteomic analyses for embryonic skeletal development in sheep had been little involved in the past research. In this study, we explored differential abundance proteins during embryonic skeletal muscle development by the tandem mass tags (TMT) and performed a protein profile analyses in the longissimus dorsi of Chinese merino sheep at embryonic ages Day85 (D85N), Day105 (D105N) and Day135 (D135N). 5,520 proteins in sheep embryonic skeletal muscle were identified, and 1,316 of them were differential abundance (fold change ≥1.5 and *p*-value < 0.05). After the KEGG enrichment analyses, these differential abundance proteins were significant enriched in the protein binding, muscle contraction and energy metabolism pathways. After validation of the protein quantification with the parallel reaction monitoring (PRM), 41% (16/39) significant abundance proteins were validated, which was similar to the results of protein quantification with TMT. All results indicated that D85N to D105N was the stage of embryonic muscle fibers proliferation, while D105N to D135N was the stage of their hypertrophy. These findings provided a deeper understanding of the function and rules of proteins in different phases of sheep embryonic skeletal muscle growth and development.

## Introduction

In recent years, study on embryonic skeletal muscle formation or myogenesis was paid more and more attention. The growth and development of skeletal muscle in different embryonic stages are the main contributor to mass and therefore directly related to meat production^1^. The embryonic skeletal muscle of vertebrate is derived from the progenitors originating in the somites and migrating into the limbs, where cells increase and disintegrate into different types of cells: muscle or endothelial^2,3^. The structure of mature skeletal muscle is determined during embryogenesis, which is an important process in skeletal muscle development and involve in a variety of metabolic functions in organisms^4,5^. The process of skeletal muscle development can be divided into two distinct phases. In first phase, the number of skeletal muscle cells are increasing at early embryo. In the second, the cells are hypertrophic at later stage of growth and the number of cells are set at the phases of cell hyperplasia^6^, in which some proteins and transcription factors, as regulators^7–11^, play a cardinal role in the development process of embryonic skeletal muscle and muscle differentiation.

Proteomics is a powerful tool for analyses of protein expression file and emergence of some novel function. At present, the mechanism of sheep embryonic skeletal muscle development is not clear. A few of studies on proteomics of sheep muscle hypertrophy were reported^12–16^. In order to illuminate the real mechanism of embryonic skeletal muscle growth, development and maturation, a wide and deep analyses of differential abundance proteins is necessary. Here we constructed the protein profile of the longissimus dorsi of Chinese merino sheep at different embryonic ages and explored the function of differential abundance proteins in them.

## Results

### The profile of protein

**Figure 1:**
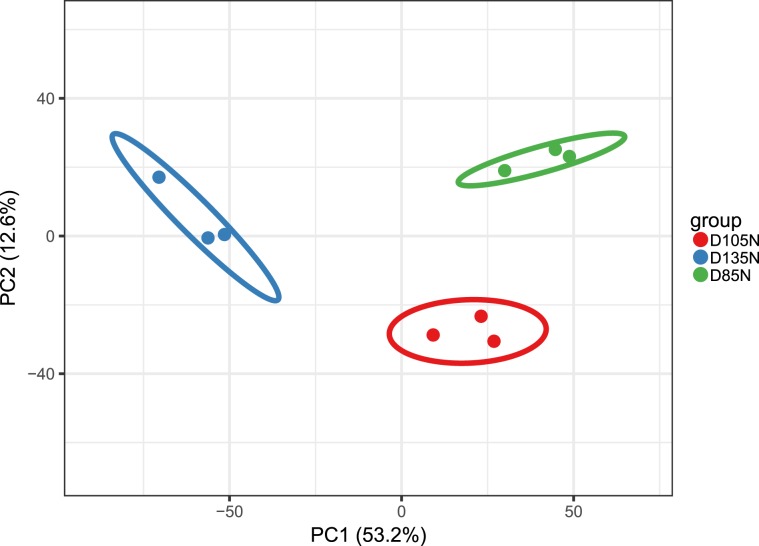
Principal component analyses. PCA plots of the two components of all samples. The fraction of the total variance explained is reported on each individual axis between parentheses.

**Figure 2:**
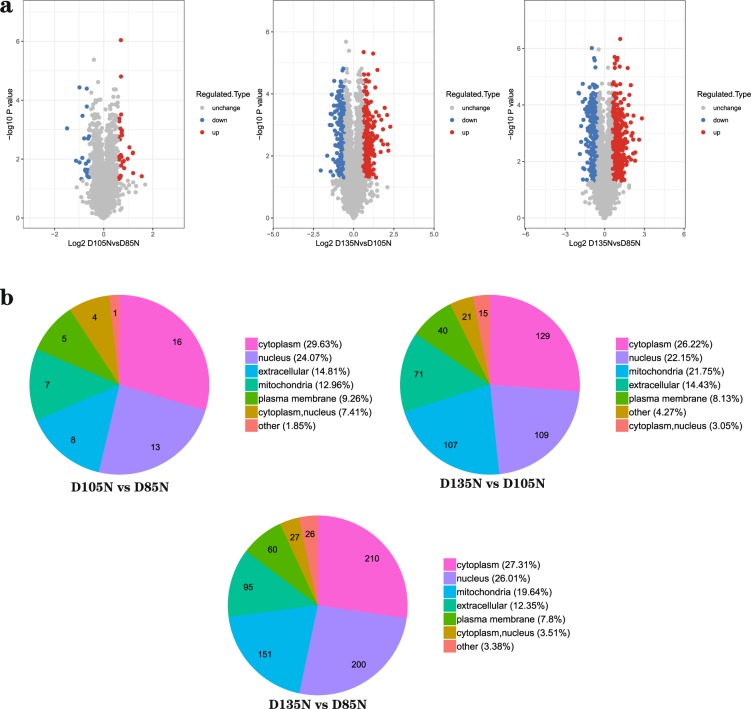
Differential abundance proteins in three comparable groups. (**a**) Volcano plot of the differential abundance proteins. The expression of the differential abundance proteins in three comparable groups. Red point indicates up-regulated, Blue point indicates down-regulated and Grey point indicates unchanged. (**b**) Subcellular localization chart of differential abundance proteins. Each color represents a subcellular location with different percentages.

The principal component analyses (PCA) was used for estimated the repeatability of the quantitative proteins and all results showed the repeatability was ideal (Fig. 1). In view of PCA results, all samples were distinguished clearly into three major categories and the ideal repeatability among the each pair of three repeated samples was emerged. In this study, the most spectra results had a first-order mass error less than 10ppm, which indicated that the mass spectrometer’s mass accuracy was normal. After the analyses of secondary spectrum data, the secondary spectrogram number 936,413 were obtained by mass spectrometry and 222,845 of them were available effective. The spectrum utilization rate was 23.8% (222,845/936,413). The total peptides of 111,477 were identified by spectral analyses and 99,974 peptides of them were specific. Then 6,391 proteins were identified. 5,520 of them were quantified (the detail of the quantitation proteins as Supplementary Table S1) and 1,316/5,520 were differential abundance proteins (the detail of differential abundance proteins as Supplementary Table S2). Results of comparable analyses showed that, in differential abundance proteins, 33 proteins were up-regulated and 21 were down-regulated in D105N vs. D85N, 272 proteins up-regulated and 220 down-regulated in D135N vs. D105N, 419 proteins up-regulated and 351 down-regulated in D135N vs. D85N. The subcellular localization results of differential abundance proteins were significant enriched in cytoplasm (Fig. 2).

### Function analyses of differential abundance proteins

**Figure 3:**
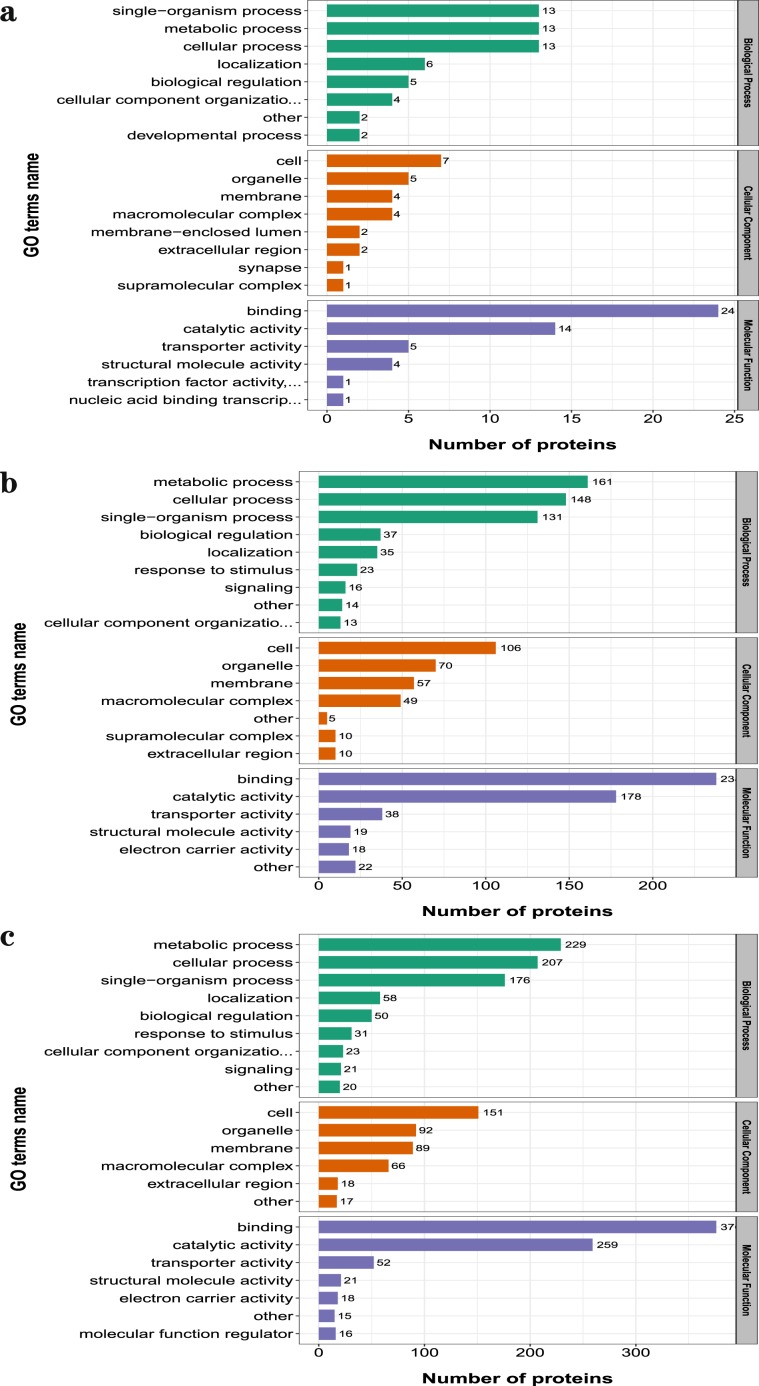
The GO secondary annotation of differential abundance proteins. Proteins as classified into three main categories by GO analyses, green indicates biological process, orange indicates cellular component and purple indicates molecular function. (**a**) D105N vs. D85N, (**b**) D135N vs. D105N and (**c**) D135N vs. D85N. Y-axis indicates the GO secondary annotation names, X-axis indicates the number of GO secondary annotation proteins.

**Figure 4:**
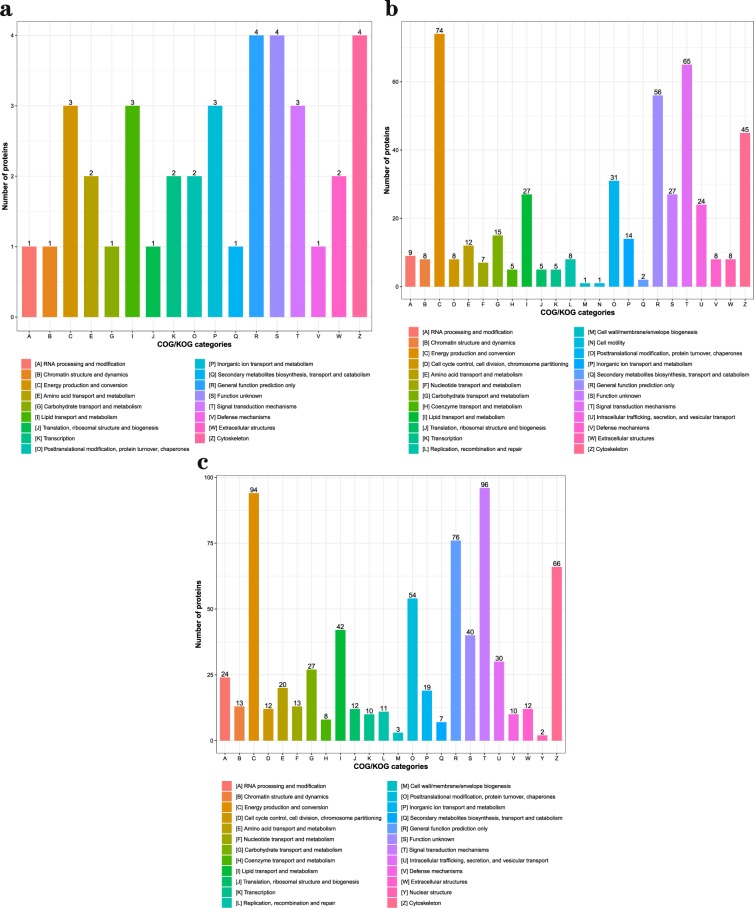
The functional classification chart of differential abundance proteins. Orthologous proteins as classified into different functions. (**a**) D105N vs. D85N, (**b**) D135N vs. D105N and (**c**) D135N vs. D85N. Y-axis indicates the number of proteins and X-axis indicates the categories of orthologous proteins.

**Figure 5:**
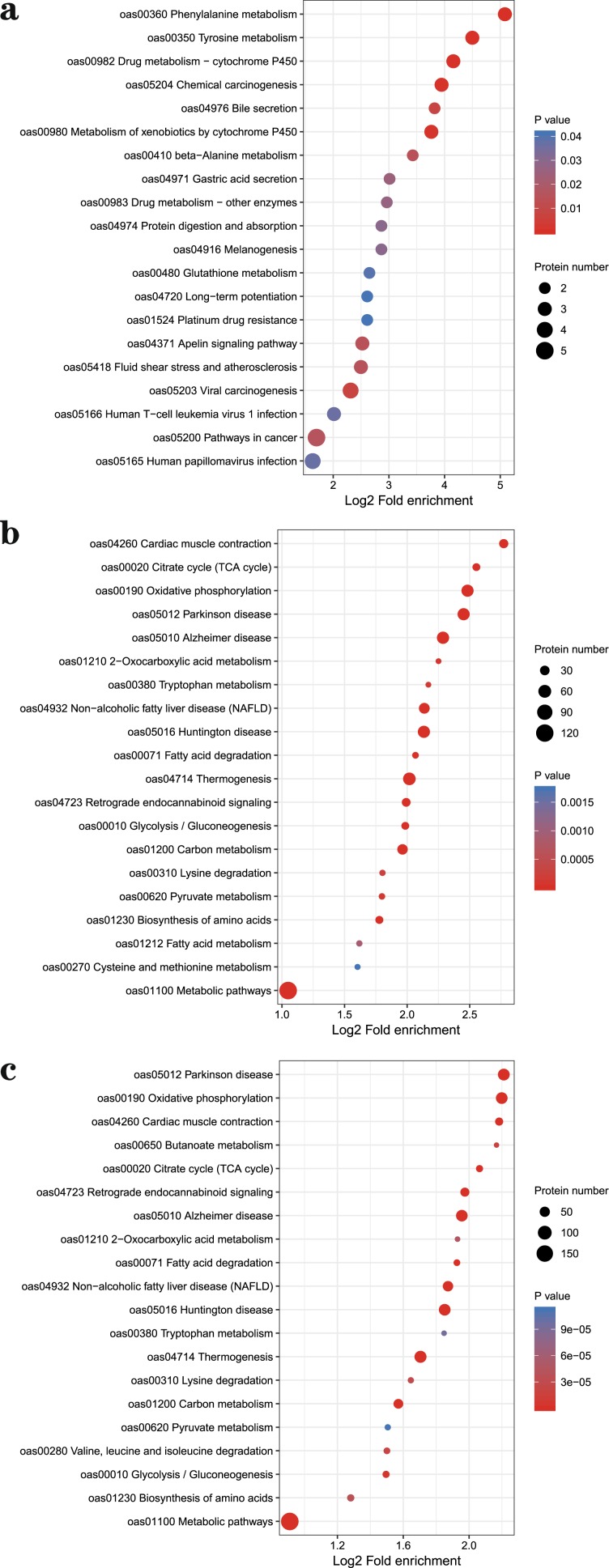
The KEGG enrichment of differential abundance proteins. Proteins enriched into different pathways by KEGG analyses. (**a**) D105N vs. D85N, (**b**) D135N vs. D105N and (**c**) D135N vs. D85N. Y-axis indicates the pathway names, X-axis indicates the factors that the number of differential abundance proteins enriched in pathways. And the size and color of the bubble indicates the number and significant characters of differential abundance proteins that enriched in pathways, red point indicates significant and blue point indicates not significant.

After Go analyses, in three comparable groups, the differential abundance proteins were mainly involved in metabolic and cellular biological processes, binding and catalytic activation molecular functions in cells (Fig. 3). To deeply analyze the differential abundance proteins, the functional classification of clusters of orthologous group protein (COG) (Fig. 4) and KEGG pathways enrichment (Fig. 5) were used to regard the function and characteristic. Interestingly, the results of COG functional classification in differential abundance proteins showed that the cytoskeletal proteins were mainly expressed in D105N vs. D85N. And they played an important role in regulating the contraction and maintaining the morphology of skeletal muscle cells at the early development phase^17^. While, the energy production and conversion proteins were significant expressed in D135N vs. D105N, the signal transduction and the energy production and conversion were expressed in D135N vs. D85N. And all results of function analyses indicated that the rules of the differential abundance proteins were consistent with the growth and development of skeletal muscle. And all analysing results indicated that the number of embryonic skeletal muscle fibres were increased mainly in D85N to D105N. But in D105N to D135N, the embryonic skeletal muscle fibres were hypertrophic with a higher energy metabolism. While the regulatory proteins of muscle contraction were up-expressed in D135N vs. D85N.

### PRM validation of TMT data

The results of the validation protein fragment ion peptides indicated that 16 proteins were validated and 11 of them were quantified with two unique peptides (Table 1). After analyses, the results demonstrated that the quantitation ratios of TMT and those of PRM were consistent each other (Table 2). So the reliability of the TMT quantitation data and the advantages of the PRM technology were notable. (the detail validation of the PRM as Supplementary Table S3)

**Table 1:** The PRM quantitation proteins.

Protein Accession	Protein description	Regulated Type	Peptides
XP_012041646.1	PREDICTED: myosin light chain 4 [*Ovis aries*]	Down	EAFSLFDR IAYGQCGDVLR
XP_014958377.1	PREDICTED: insulin-like growth factor II isoform X2 [*Ovis aries*]	Down	SCDLALLETYCAAPAK
XP_004018449.1	“PREDICTED: troponin C, slow skeletal and cardiac muscles [*Ovis aries*]”	Up	SEEELSDLFR
NP_001009484.1	cofilin-1 [*Ovis aries*]	Down	EILVGDVGQTVDDPYATFVK
NP_001155363.1	“fumarate hydratase, mitochondrial [*Ovis aries*]”	Up	IEYDTFGELK AAAEVNQDYGLDPK
XP_014955319.1	PREDICTED: microtubule-associated protein RP/EB family member 1 [*Ovis aries*]	Down	LEHEYIQNFK
NP_001135426.1	myosin regulatory light chain 2, ventricular/cardiac muscle isoform [*Ovis aries*]	Up	GADPEETILNAFK
XP_014948822.1	PREDICTED: titin isoform X5 [*Ovis aries*]	Up	IASLEIPLAK GSPVIQVTWFK
XP_004015439.1	PREDICTED: myosin-binding protein C, fast-type isoform X1 [*Ovis aries*]	Up	QLEVLQDIADLTVK TSDFDTVFFVR
XP_014954142.1	PREDICTED: LOW QUALITY PROTEIN: myosin-3 isoform X1 [*Ovis aries*]	Down	VEDEQTLGLQFQK LELDDLGSNVESVSK
XP_004012685.1	PREDICTED: very long-chain specific acyl-CoA dehydrogenase, mitochondrial [*Ovis aries*]	Up	VPAENVLGEVGGGFK GQLTTDQVFPYPSVLNEDQTQFLK
XP_011957551.2	PREDICTED: LOW QUALITY PROTEIN: alpha-actinin-3 isoform X1 [*Ovis aries*]	Up	GYEDWLLSEIR VGWEQLLTSIAR
XP_004008702.1	PREDICTED: fibrillin-2 [*Ovis aries*]	Down	GTCWLNIQDNR GFSLDATGLNCEDVDECDGNHR
XP_004010374.1	PREDICTED: myosin-7 [*Ovis aries*]	Up	LLGSLDIDHNQYR DFELNALNAR
NP_001177319.1	glyceraldehyde-3-phosphate dehydrogenase [*Ovis aries*]	Up	AITIFQER VPTPNVSVVDLTCR
NP_001159670.1	four and a half LIM domains protein 1 [*Ovis aries*]	Up	DCFTCSNCK FTAVEDQYYCVDCYK

**Table 2:** Comparable quantification results of TMT and PRM.

Protein Accession	Protein description	Comparable group	TMT quantification ratios	PRM quantification ratios
XP_012041646.1	PREDICTED: myosin light chain 4 [*Ovis aries*]	a	0.74	0.49
		b	0.65	0.61
		c	0.48	0.30
XP_014958377.1	PREDICTED: insulin-like growth factor II isoform X2 [*Ovis aries*]	a	0.92	0.67
		b	0.65	0.42
		c	0.60	0.28
XP_004015439.1	“PREDICTED: myosin-binding protein C, fast-type isoform X1 [*Ovis aries*]”	a	1.96	9.15
		b	1.06	1.86
		c	2.07	17.05
XP_004018449.1	“PREDICTED: troponin C, slow skeletal and cardiac muscles [*Ovis aries*]”	a	0.70	0.64
		b	2.40	4.62
		c	1.68	2.94
NP_001155363.1	“fumarate hydratase, mitochondrial [*Ovis aries*]”	a	1.08	0.86
		b	1.66	1.46
		c	1.79	1.25
XP_014955319.1	PREDICTED: microtubule-associated protein RP/EB family member 1	a	0.83	0.54
		b	0.76	0.43
		c	0.63	0.23
NP_001135426.1	“myosin regulatory light chain 2, ventricular/cardiac muscle isoform [*Ovis aries*]”	a	0.65	0.37
		b	2.05	3.27
		c	1.33	1.22
XP_014948822.1	PREDICTED: titin isoform X5 [*Ovis aries*]	a	1.11	0.68
		b	1.47	1.66
		c	1.63	1.12
XP_004015439.1	“PREDICTED: myosin-binding protein C, fast-type isoform X1 [*Ovis aries*]”	a	1.96	9.15
		b	1.06	1.86
		c	2.07	17.05
XP_014954142.1	PREDICTED: LOW QUALITY PROTEIN: myosin-3 isoform X1 [*Ovis aries*]	a	0.79	0.52
		b	0.52	0.25
		c	0.41	0.13
XP_004012685.1	“PREDICTED: very long-chain specific acyl-CoA dehydrogenase, mitochondrial [*Ovis aries*]”	a	1.08	0.89
		b	2.08	2.68
		c	2.25	2.39
XP_011957551.2	PREDICTED: LOW QUALITY PROTEIN: alpha-actinin-3 isoform X1 [*Ovis arie****s*]**	a	1.66	7.50
		b	1.35	2.82
		c	2.24	21.18
XP_004008702.1	PREDICTED: fibrillin-2	a	0.92	0.73
		b	0.63	0.36
		c	0.58	0.27
XP_004010374.1	PREDICTED: myosin-7 [*Ovis aries*]	a	0.91	0.68
		b	2.55	4.95
		c	2.33	3.35
NP_001177319.1	glyceraldehyde-3-phosphate dehydrogenase [*Ovis aries*]	a	1.11	0.93
		b	1.66	1.78
		c	1.83	1.66
NP_001159670.1	four and a half LIM domains protein 1 [*Ovis aries*]	a	1.16	0.96
		b	2.05	2.09
		c	2.36	2.02

(a) D105N vs. D85N, (b) D135N vs. D105N and (c) D135N vs. D85N. The ratios of TMT and PRM quantification are the average protein quantitation at one stage to another one such as the a, b and c. (The detail can be found in Supplementary Table S3).

## Discussion

The embryonic phase is important for skeletal muscle development and growth^18^. During this phase, the muscle fibres are formed and proliferated at early embryonic stage and hypertrophic at late stage^19,20^, which has great influence on production of meat mass^21^. In this paper, the protein profile of the embryonic skeletal muscle in sheep was established by using the TMT^22,23^ and validated by PRM technology^24,25^. TMT is widely implemented in the higher throughput proteomics, and PRM can be used to quantify protein abundance without specific antibodies and detect specific predetermined with known fragmentation characteristics more accurately in complex backgrounds than the traditional immunoassays^26–30^. And the results of TMT quantitation were entirely validated by PRM (Table 1). Finally, a total number of 5,520 proteins were identified and 1,316 of them were differential abundance proteins, which will help us to unveil the mechanisms of embryonic skeletal muscle development and growth of sheep.

After deep analyses of these data, we found that the cytoskeletal proteins and the energy production and conversion proteins were mainly expressed in D105N vs. D85N and D135N vs. D105N respectively (Fig. 4). And the results of GO and KEGG analyses revealed that these proteins were enriched in the protein binding, muscle contraction and energy metabolism pathways which took part in the regulation in the process of embryonic skeletal muscle development and growth of sheep^31–35^. And the cytoskeletal proteins with higher abundance in stage of muscle fibers proliferation and the energy metabolism played a major role in the embryonic muscle hypertrophy^13,36^, this indicated that D85N to D105N was the stage of embryonic muscle fibers proliferation and D105N to D135N was hypertrophy.

The metabolic pathway was very significant in the process of embryonic skeletal muscle development. And the metabolic pathway decided the muscle fibers proliferation and hypertrophy and regulated the protein differentiation and cell cycle^37^. Meanwhile, a few of reports showed that energy metabolism significantly regulated the myoblast to be in hypertrophy, but not in the cycle of proliferation withdrew from the cycle of cell proliferation and turned into muscle hypertrophy by precisely regulating the protein activity of myoblast cycle. The myoblast cycle was regulated by changing the phosphorylation levels of their cycle regulation proteins under the different metabolic condition^38,39^.

In both of our study (Fig. 5) and other reports, all results showed that the metabolic and oxidative phosphorylation pathways were significantly associated with the development of muscle^40–42^. In a word, the metabolism provided not only energy in whole embryonic skeletal muscle development but also fine modulation of embryonic muscle development styles^43–45^. As to the protein profile constructed in this study, it will be certainly an important basis for deep elucidation of the mechanisms of sheep embryonic skeletal muscle development and growth.

## Methods

### Experimental design and samples preparation

Embryonic skeletal muscle samples were taken from different development phases of foetal (the detail can be found in Supplementary Information S1). And on the basis of reports before^46^, the pregnant of female sheep were selected on D85N, D105N and D135N for caesarean section. The foetuses with similar embryonic ages were taken out and the longissimus dorsi was collected and there were three biological repeats per time phases (no mixing between per time phase samples). Furthermore, three comparable groups such as the D105N vs. D85N, D135N vs. D105N and D135N vs. D85N were set to study. Finally, these samples were immediately washed clear with PBS (pH7.2), then frozen in liquid nitrogen. All of samples were kept at −80 °C until further analyses.

### Animal welfare disclaimer

The works herein described was conducted according to relevant animal care protocols, which were approved by the relevant guidelines and regulations of the Ministry of Agriculture of the People’s Republic of China (Beijing, China). Furthermore, in this study, all use and handing of experimental animals were made to minimize suffering.

### Protein extraction and TMT labeling

Samples used for extraction of total proteins in each stage D85N, D105N and D135N were prepared according to the protocol as follows. The samples were taken out at −80 °C, and an appropriate amount of tissue sample was weighed into a liquid nitrogen pre-cooled mortar, and liquid nitrogen was sufficiently ground to a powder. Each group of samples were separately added to 4 volumes of lysis buffer (8 mol urea, 1% protease inhibitor and 2 mmol EDTA) and sonicated. After centrifugation at 12,000 g for 10 min at 4 °C, the cell debris were removed, the supernatant was transferred to a new centrifuge tube, and the protein concentration was determined using the BCA kit (Beyotime Biotechnology, China). Post addition of the dithiothreitol into the protein solution to a final concentration of 5 mmol, digestion was performed at 56 °C for 30 min. Then the iodoacetamide was added to a final concentration of 11 mmol and incubated for 15 min at room temperature in dark condition. Finally, the urea concentration in sample was diluted to less than 2 mol. Trypsin (1 μg/μl) was added at a mass ratio of 1:50 (pancreatin: protein sample, V/V) and digested overnight at 37 °C. Trypsin was added at a mass ratio of 1:100 (pancreatin: protein sample, V/V) and continued to digest for 4 h.

After trypsin digestion, the peptides were desalted by Strata X C18 SPE column (Phenomenex) and vacuum-dried, all stage groups were reconstituted in 0.5 mol TEAB and labelled with three labels according to the manufacturer’s direction for TMT kit (Thermo, USA). Briefly, one unit of the TMT reagent was thawed and reconstituted in acetonitrile. Then, the peptides mixture with an equal molar ratio were incubated at room temperature for 2 h. These were combined and desalted. Finally, the TMT labelling peptide segments were completed by vacuum centrifugal drying.

### First-dimensional separation of TMT-labeled peptides

The tryptic peptides were fractionated into several fractions by high pH reverse-phase HPLC with the Agilent 300 Extend C18 column (5 μmol particles, 4.6 mm ID, 250 mm length, Agilent Technologies, Palo Alto, CA). Based on the fractional gradient 8–32% acetonitrile, the peptides were separated into 60 fractions and each of them was incubated at pH6.0 for 60 min specifically. Finally, the peptides were combined into 9 fractions (final fraction 1 = 1, 10, 19, 28, 37, 46, 55; final fraction 2 = 2, 11, 20, 29, 38, 47, 56; final fraction 3 = 3, 12, 21, 30, 39, 48, 57; …… final fraction 9 = 9, 18, 27, 36, 45, 54.) and then they were dried by vacuum centrifuging for subsequent operations.

### LC-MS/MS analyses

The peptides in each fractions were dissolved in liquid phase A phase and analysed by using the EASY-nLC 1200 ultra-high performance liquid system interfaced with a reversed-phase analytical column (15 cm length, 75 μmol ID). The liquid phase A is an aqueous solution containing 0.1% formic acid and 2% acetonitrile. While the liquid phase B is an aqueous solution containing 0.1% formic acid and 90% acetonitrile. The liquid phase gradient settings were in 8–16% B for about 30 min, 16–30% B for 15 min, 30–80% B for 2 min and 80% B for 3 min step by step. The flow rate maintained at 400 nl/min. Then, the peptides were separated by an ultra-high performance liquid phase system and they were injected into an NSI ion source for ionization and analysed by Orbitrap Lumos™ mass spectrometry. The ion source voltage was set to 2.0 kV and the peptide precursor and its secondary fragments were detected and analysed using the high-resolution Orbitrap.

The primary mass spectrometer scan range was set to 350~1,550 m/z, and the scan resolution was set to 60,000. The secondary mass spectrometry scan range had a fixed starting point of 100 m/z, and the secondary scan resolution was set to 15,000. In the end, the data acquisition mode used a data-dependent scanning (DDA) program. After the first-stage scanning, the first 20 peptides with highest signal intensity were selected to enter the high-energy collision-induced dissociation (HCD). Subsequently, 32% of the energy was used for fragmentation and also performed in peptides. In order to improve the effective utilization of mass spectrometry and to avoid parent ions. In this analyses, the automatic gain control (AGC) was set to 50,000, the signal threshold 50,000 ions/s, the maximum injection time 70 ms and the dynamic exclusion time of tandem mass spectrometry 30 s.

### Data analyses

All proteins in 9 fractions were analyzed and compared with Maxquant engine v.1.5.2.8 (http://www.maxquant.org/) and tandem mass spectra were searched against the NCBI *Ovis aries* Oar_v4.0 (https://www.ncbi.nlm.nih.gov/genome/? term = *Ovis + aries* 43,036 sequences) database. Meanwhile, in order to eliminate the influence of contaminating proteins in the identification results, the reverse decoy database to calculate the false discovery rate (FDR) caused by random matching and common pollution database were concatenated. And the Trypsin/P was specified as cleavage enzyme allowing up to 2 missing cleavages. The mass tolerance for precursor ions was set as 20ppm in the first search and 5ppm in the main search. But the mass tolerance for fragment ions was set as 0.02 Da. And the quantitative method was set as TMT-6plex, the FDR for protein identification was set as 1%. The differential abundance proteins were calculated by the mean of the three repeats to another mean of the three repeats. Then T-tests between two-samples were used to compare differential abundance proteins. A significance level (*P*-value) of 0.05 was used for statistical analyses any time. Post the statistical analyses, the fold change 1.2, 1.3, 1.5 and 2 were set for the quantitation proteins. Based on the standard which the ratio of quantitative protein to differential abundance protein is less than 30% (Table 3), the fold change ≥ 1.5 and *p*-value < 0.05 were considered to be differential abundance proteins. For TMT quantification, the ratios of the TMT reporter ion intensities in MS/MS spectra (126–131 m/z) from raw data sets were used to calculate fold changes among samples. For each sample, the quantification was medium-normalized at peptide level to center the distribution of quantitative values. Protein quantitation was then calculated as the median ratio of corresponding unique peptide for given protein. The analyses of each replicate was performed only once.

**Table 3:** Differential fold change abundance proteins summary.

Compare group	Regulated type	Fold change > 1.2	Fold change > 1.3	Fold change > 1.5	Fold change > 2
D105N vs. D85N	all-regulated	391	190	54	7
D135N vs. D105N	all-regulated	1521	1066	492	90
D135N vs. D85N	all-regulated	1914	1430	770	219

Based on the standard which the ratio of quantitative protein to differential abundance protein is less than 30%, the fold change ≥ 1.5 and *p*-value < 0.05 were considered to be differential abundance proteins.

### Bioinformatics analyses

All identified proteins were annotated and classified according to the protein sequence-based algorithm software InterProScan v.5.14–53.0 (http://www.ebi.ac.uk/interpro/), which predicted the GO function of proteins and classifies them based on cellular composition, molecular function and biological process. Then protein pathways were clustered by using the KEGG pathway database to annotate. At first, the submitted proteins were annotated using the KEGG online service tool KAAS v.2.0 (http://www.genome.jp/kaas-bin/kaas_main), then the annotated proteins were matched to the corresponding pathway in the database by the KEGG mapper V2.5 (http://www.kegg.jp/kegg/mapper.html). Furthermore, the Fischer’s exact double-end test (Fisher’s exact test) was used to probe differential abundance proteins as a background for enrichment analyses by the Perl module v.1.31 (https://metacpan.org/pod/Text::NSP::Measures::2D::Fisher). The clustering relationships of the differential abundance proteins were visualized by using the Heatmap which can be drawn by the R package of heatmap2 and Gplots v.2.0.3 (https://cran.r-project.org/web/packages/cluster/). And the Wolfpsort v.0.2 (http://www.genscript.com/psort/wolf_psort.html) software was used to perform subcellular structural localization prediction and classification statistics for differential abundance proteins.

### Quantitative validation based on targeted proteomics

The extraction, digestion and mass spectrometry of validation proteins were same as before description and each sample of 1.5 µg hydrolysed peptides was validated. The results of MS data were processed using Skyline v. 3.6. Indexes for Peptide were set as: Trypsin digestion [KR/P], the max missed cleavage 2 and the peptide length 8–25 aa that the max variable modification of carbamidomethyl on Cys and oxidation on Met was set to 3. For transition sets, precursor charges were set as 2 or 3, ion charges as 1 or 2 and ion types as b, y and p. But the ions of product were set as from ion 3 to the last ion, and the value of ion matching tolerance was set as 0.02 Da. The all data of analyses were visualized by Skyline^47^.

In our study, to validate the TMT quantitation results by using the PRM. 40 differential abundance proteins were randomly selected from three comparable groups (the detail validation of the PRM as Supplementary Table S3).

*(The current research is based on a small amount of animals and may be considered as preliminary).

## Supplementary information

Supplementary Table S1 the detail of the quantitation proteins.

Supplementary Table S2 the detail of differential abundance proteins.

Supplementary Table S3 the detail validation of the PRM.

Supplementary Information S1 the detail of samples preparation.

## References

[1] Guller I, Russell AP (2010). MicroRNAs in skeletal muscle: their role and regulation in development, disease and function. J. Physiol..

[2] Buckingham M (2002). 15 The formation of skeletal muscle: from somite to hand. J. Anat..

[3] Gabrielle Kardon JKC, Clifford JT (2002). Local Extrinsic Signals Determine Muscle and Endothelial Cell Fate and Patterning in the Vertebrate Limb. Developmental Cell.

[4] Bentzinger, C. F., Wang, Y. X. & Rudnicki, M. A. Building muscle: molecular regulation of myogenesis. *Cold Spring Harb Perspect Biol* **4** (2012). 10.1101/cshperspect.a008342 PMC3281568 22300977

[5] Tajbakhsh S (2010). Skeletal muscle stem cells in developmental versus regenerative myogenesis. J. Intern. Med..

[6] White RB, Biérinx AS, Gnocchi VF, Zammit PS (2010). Dynamics of muscle fibre growth during postnatal mouse development. Bmc Developmental Biol..

[7] Buckingham M (2007). Skeletal muscle progenitor cells and the role of Pax genes. C. R. Biol..

[8] Buckingham M, Relaix F (2015). PAX3 and PAX7 as upstream regulators of myogenesis. Semin. Cell Dev. Biol..

[9] Sabourin LA, Rudnicki MA (2010). The molecular regulation of myogenesis. Clin. Genet..

[10] Murphy M, Kardon G (2011). Origin of vertebrate limb muscle: the role of progenitor and myoblast populations. Curr. Top. Dev. Biol..

[11] Hernandez-Hernandez JM, Garcia-Gonzalez EG, Brun CE, Rudnicki MA (2017). The myogenic regulatory factors, determinants of muscle development, cell identity and regeneration. Semin. Cell Dev. Biol..

[12] Ferreira AM (2017). The sheep (Ovis aries) muscle proteome: Decoding the mechanisms of tolerance to Seasonal Weight Loss using label-free proteomics. J. Proteom..

[13] Hamelin M (2006). Proteomic analysis of ovine muscle hypertrophy. J. Anim. Sci..

[14] Miller B (2019). Ovine liver proteome: Assessing mechanisms of seasonal weight loss tolerance between Merino and Damara sheep. J. Proteom..

[15] Almeida AM (2016). The Effect of Weight Loss on the Muscle Proteome in the Damara, Dorper and Australian Merino Ovine Breeds. PLoS One.

[16] Yu T-Y, Morton JD, Clerens S, Dyer JM (2015). In-depth characterisation of the lamb meat proteome from longissimus lumborum. EuPA Open. Proteom..

[17] Golson ML, Sanger JM, Sanger JW (2010). Inhibitors arrest myofibrillogenesis in skeletal muscle cells at early stages of assembly. Cytoskeleton.

[18] Ouyang H (2017). Proteomic Analysis of Chicken Skeletal Muscle during Embryonic Development. Front. Physiol..

[19] Ashmore CR, Robinson DW, Rattray P, Doerr L (1972). Biphasic development of muscle fibers in the fetal lamb. Exp. Neurol..

[20] Wigmore PMSNC (1983). Muscle development in large and small pig fetuses. J. Anat..

[21] Voillet V (2018). Integrated Analysis of Proteomic and Transcriptomic Data Highlights Late Fetal Muscle Maturation Process. Mol. Cell. Proteom. Mcp.

[22] Chen M, Zhu A, Storey KB (2016). Comparative phosphoproteomic analysis of intestinal phosphorylated proteins in active versus aestivating sea cucumbers. J. Proteom..

[23] Iwata H (2016). PARP9 and PARP14 cross-regulate macrophage activation via STAT1 ADP-ribosylation. Nat. Commun..

[24] Gallien S, Bourmaud A, Kim SY, Domon B (2014). Technical considerations for large-scale parallel reaction monitoring analysis. J. Proteom..

[25] Amelia C (2012). Parallel Reaction Monitoring for High Resolution and High Mass Accuracy Quantitative, Targeted Proteomics. Mol. Cell. Proteom..

[26] Marx V (2012). Targeted proteomics. Nat. Methods.

[27] Picotti P, Aebersold R (2012). Selected reaction monitoring-based proteomics: workflows, potential, pitfalls and future directions. Nat. Methods.

[28] Doerr A (2012). Targeting with PRM. Nat. Methods.

[29] Ronsein GE (2015). Parallel reaction monitoring (PRM) and selected reaction monitoring (SRM) exhibit comparable linearity, dynamic range and precision for targeted quantitative HDL proteomics. J. Proteom..

[30] Schilling B (2015). Multiplexed, Scheduled, High-Resolution Parallel Reaction Monitoring on a Full Scan QqTOF Instrument with Integrated Data-Dependent and Targeted Mass Spectrometric Workflows. Anal. Chem..

[31] Liu S, Han W, Jiang S, Zhao C, Wu C (2016). Integrative transcriptomics and proteomics analysis of longissimus dorsi muscles of Canadian double-muscled Large White pigs. Gene.

[32] Timson DJ, Trayer HR, Smith KJ, Trayer IP (1999). Size and charge requirements for kinetic modulation and actin binding by alkali 1-type myosin essential light chains. J. Biol. Chem..

[33] Grabarek Z (2006). Structural basis for diversity of the EF-hand calcium-binding proteins. J. Mol. Biol..

[34] Gerrard DE, Okamura CS, Ranalletta MA, Grant AL (1998). Developmental expression and location of IGF-I and IGF-II mRNA and protein in skeletal muscle. J. Anim. Sci..

[35] Picard B, Lefaucheur L, Berri CC, Duclos MJ (2002). Muscle fibre ontogenesis in farm animal species. Reprod. Nutr. Dev..

[36] Stromer MH (1998). The cytoskeleton in skeletal, cardiac and smooth muscle cells. Histology Histopathology.

[37] Sandri M (2008). Signaling in muscle atrophy and hypertrophy. Physiol..

[38] Braun T, Gautel M (2011). Transcriptional mechanisms regulating skeletal muscle differentiation, growth and homeostasis. Nat. Rev. Mol. Cell Biol..

[39] Chakravarthy MV, Spangenburg EE, Booth FW (2001). Culture in low levels of oxygen enhances *in vitro* proliferation potential of satellite cells from old skeletal muscles. Cell. Mol. Life Sci. Cmls.

[40] Heeley DH (2013). Phosphorylation of tropomyosin in striated muscle. J. Muscle Res. Cell Motil..

[41] Deshmukh AS (2015). Deep proteomics of mouse skeletal muscle enables quantitation of protein isoforms, metabolic pathways and transcription factors. Mol. Cell. Proteom..

[42] Yang S (2018). Parallel comparative proteomics and phosphoproteomics reveal that cattle myostatin regulates phosphorylation of key enzymes in glycogen metabolism and glycolysis pathway. Oncotarget.

[43] Murgia M (2015). Single muscle fiber proteomics reveals unexpected mitochondrial specialization. EMBO Rep..

[44] Sayd T, Mera T, Martin V, Laville E (1998). Spatial distribution of myosin heavy chain isoforms and lactate dehydrogenase M4 in the limb musculature of two crossbred lambs. Comp. Biochem. Physiol. B Biochem Mol. Biol..

[45] Liu J (2016). Protein Profiles for Muscle Development and Intramuscular Fat Accumulation at Different Post-Hatching Ages in Chickens. PLoS One.

[46] Peñagaricano F, Xin W, Rosa GJ, Radunz AE, Khatib H (2014). Maternal nutrition induces gene expression changes in fetal muscle and adipose tissues in sheep. Bmc Genomics.

[47] Brendan ML (2010). Skyline: an open source document editor for creating and analyzing targeted proteomics experiments. Bioinforma..

