# Supplementary material for: Proteomic analyses of sheep (ovis aries) embryonic skeletal muscle
Source: Sci Rep. 2020 Feb 4;10:1750. doi: 10.1038/s41598-020-58349-0 (PMC7000794; doi:10.1038/s41598-020-58349-0)
Supplement: Supplementary file 4 — Supplementary Information S1 the detail of samples preparation. [file 41598_2020_58349_MOESM4_ESM.pdf]

Proteomic analyses of sheep (*ovis aries*) embryonic skeletal muscle

Xinyue Wang¶, Tianpei Shi¶, Zhida Zhao, Haobin Hou, Li Zhang*
Institute of Animal Science, Chinese Academy of Agricultural Sciences, (CAAS) Beijing, China

Supplementary Table S1. The detail of artificial insemination experiment and feeding condition.

In this experiment, the adult Chinese merino sheep with same conditions and body weight 55 to 60kg were taken from Xinjiang province. the feeding ratio of sheep to the hay and silage was 1:3 and the amount of concentrated feed in morning and evening were 0.6kg/day and 0.3kg/day respectively. And the sheep were allowed to freely feed forage and ensured to drink water (this feeding condition was also implemented in the pregnant ewes). Based on this feeding condition, the 0.8kg forage per day, one egg piece/day (mix or douse) and carrot 2kg/ piece/day were supplemented to the rams. Meanwhile, the ewes were treated with progesterone vaginal suppository (Vetoquinol, France) for oestrus treatment in preparation for artificial insemination. And a ram was selected to mating. Meanwhile, the sperm was collected from the rams 3 or 4 times a day and the quality of semen was checked. The B-ultrasonography was used to screen the condition of the ewes and the ewes were selected with the same condition for artificial insemination. Then, we transplanted embryos from the supernumerary ovulation into recipient ewes of the same physical condition.
